# Parturient Paresis1Read before the meeting of the Wisconsin Society of Veterinary Graduates, September, 899.

**Published:** 1900-03

**Authors:** A. H. Hartwig

**Affiliations:** Watertown, Wis.


### PARTURIENT PARESIS.[1]

By A. H. Hartwig, M.D.C.,
WATERTOWN, WIS.

Since our last meeting, held at Madison, at which I was requested to take up the subject of parturient apoplexy, or, as it has been recently termed, parturient paresis, I have watched with greater interest than ever the various theories advanced as to the pathology and therapeutical treatment of this disease.

[1] Read before the meeting of the Wisconsin Society of Veterinary Graduates, September, 899.

Among the most popular of the day is that of Professor J. Schmidt, but as we really do not know the true cause of this disease, the Schmidt theory would first appear as an empirical one. Though a little skeptical on this account, and of previous disappointments, I have concluded to give it a trial, and report the following cases:

No. 1 (fatal). I was called to a five-year-old Durham cow which was in fair flesh, but a good milker. I found her in her stall at 4 P.M., lying on her sternum and not entirely inattentive to her surroundings, but would partake of food occasionally. I was informed that she had given birth to a calf the previous day. She would attempt to rise, but was unable. After a formal examination I concluded that she had a mild attack of parturient paresis, and at once applied the potassium iodide infusion according to directions, relieving her of the contents of the rectum, which was apparently normal, and, placing her in a comfortable position, I left, giving orders to apply massage. On my arrival the next morning I found her much worse, the neck arched to one side, and groaning terribly. I concluded to repeat the dose, which I did, but not quite as strong a solution. I returned about six hours later, but saw no improvement. I then injected stimulants hypodermatically, and asked the owner to report in the morning, when about eight o'clock he arrived with the hide, stating that she had died during the night.

No. 2 (recovered). This was an eight-year-old cow of native stock. I was called at ten o'clock in the evening, and found her in a basement barn with her head turned to one side and occasionally stretching out all four limbs and groaning. She was entirely unconscious, temperature $98\frac{1}{2}°$; though the weather was warm her skin was cold and shrivelled, her mouth was cold and the tongue paralyzed; the eyes were dim, and when touched with the finger she would not attempt to close them. When examining the udder I found a slight swelling in one quarter. Upon inquiring how long since the cow had come in, I was informed that her calf was eight weeks old, which was shown to me. I could not doubt the man's word, as I really believe he was sincere in his statement. You can imagine my astonishment to see typical symptoms of parturient paresis eight weeks after parturition. Not trusting my first conclusion, I again made a thorough examination, with the same results. On account of the existing symptoms of mastitis I was not warranted to apply the potassium iodide infusion, and advised to apply friction to the exterior by means of straw wisps, which was done; also stimulating agents were applied both externally and internally, and the patient wrapped in warm blankets. The udder was frequently immersed in hot water during the night, also light massage was applied, and the udder relieved of its contents as often as possible. On my arrival the next morning my patient was resting easy and appeared much brighter. The next day she got up, and has ever since made a slow recovery, and is now giving a normal flow of milk. I am not surprised at her recovery, but have never heard of such a case eight weeks after parturition, and therefore bring it to your notice for discussion.

No. 3 (recovered). This was a very fat, short-horned cow, six years old ; had aborted the previous season, and was dry for six months prior to her period of gestation. The first symptoms of the disease were noticed about eight hours before my arrival at the place, and she was suffering with a very severe attack. I informed the owner that the case was hopeless in my opinion, but was willing to try and help her the best I could. One hundred and fifty grains of the potassium iodide were dissolved and infused according to directions ; massage applied, but no caffeine or other stimulants. This was twelve o'clock at night. I returned to the place at nine o'clock the next morning, and was informed that the cow was up. Upon reaching the stable I found her standing at her manger eating and occasionally bellowing for her calf. She soon regained her flow of milk and is again at the head of the herd.

No. 4 (recovered). Was a six-year-old red cow, not very fat, with a mild attack. I was called two hours after its first appearance. Potassium iodide was administered as before ; no stimulants. The animal made a nice recovery, and was up in six hours.

No. 5 (recovered). A medium-sized, well-fed cow, seven years old, with an attack of medium severity; treated with the potassium iodide; no stimulants ; was up after twelve hours ; did not regain her regular flow of milk until several weeks later.

No. 6 (fatal). An eight-year-old cow in fair condition, not fat, but a good milker, was found in a pasture eighteen hours after the first symptoms were manifest ; was completely paralyzed ; her general appearance resembled that of a corpse; skin and extremities very cold, eyes wide open, head and legs stretched from her ; she was not able to stir, but would utter loud, painful groans. She was brought into shelter, where we made her as comfortable as possible. Potassium iodide with mild stimulants were applied. After six hours she was resting easier, which was late at night. Upon my arrival the next morning I found her in an orchard near by, picking an occasional mouthful of grass, but was very weak.

The stimulants were kept up for several days, and she was doing nicely. When I called four days after she had a normal appetite and appeared well, but had no milk. This remained the same until the seventh day, when she stopped eating and drinking, and died several hours after. I had no opportunity for an autopsy.

No. 7. A very fat Holstein cow seven years old. I was called five hours after the trouble began. The attack was quite a severe one. Potassium iodide infused, no stimulants. The patient was up in nine hours, and made a rapid recovery.

In view of the above facts we must conclude that the potassium iodide treatment is a vast improvement over previous results, and with careful observations and experiments we can no doubt improve on it. I will add that in Case No. 1 the massage was not applied regularly as directed, which might have made some difference in the termination of the case. I have also observed that thorough cleanliness and sterilization of the infusion apparatus must be maintained. Care should also be taken that the water used is pure and thoroughly sterilized in a clean vessel, as our clients are mosly farmers who are not, as a rule, very particular in this respect.

